# Genetic polymorphism related to ethambutol outcomes and susceptibility to toxicity

**DOI:** 10.3389/fgene.2023.1118102

**Published:** 2023-04-20

**Authors:** Melisa Intan Barliana, Nadiya Nurul Afifah, Vycke Yunivita, Rovina Ruslami

**Affiliations:** ^1^ Department of Biological Pharmacy, Faculty of Pharmacy, Universitas Padjadjaran, Bandung, Indonesia; ^2^ Center of Excellence for Pharmaceutical Care Innovation, Universitas Padjadjaran, Bandung, Indonesia; ^3^ Division of Pharmacology and Therapy, Department of Biomedical Sciences, Faculty of Medicine, Universitas Padjadjaran, Bandung, Indonesia

**Keywords:** ethambutol, genetic polymorphism, personalized medicine, tuberculosis, metabolism

## Abstract

The World Health Organization (WHO) stated that ensuring access to effective and optimal treatment is a key component to eradicate tuberculosis (TB) through the End TB Strategy. Personalized medicine that depends on the genetic profile of an individual is one way to optimize treatment. It is necessary because of diverse drug responses related to the variation in human DNA, such as single-nucleotide polymorphisms (SNPs). Ethambutol (EMB) is a drug widely used as the treatment for *Mycobacterium Tuberculosis* (Mtb) and/non-tuberculous mycobacteria and has become a potential supplementary agent for a treatment regimen of multidrug-resistant (MDR) and extensively drug-resistant (XDR) TB. In human genetic polymorphism studies of anti-tuberculosis, the majority focus on rifampicin or isoniazid, which discuss polymorphisms related to their toxicity. Whereas there are few studies on EMB, the incidence of EMB toxicity is lower than that of other first-line anti-TB drugs. To facilitate personalized medicine practice, this article summarizes the genetic polymorphisms associated with alterations in the pharmacokinetic profile, resistance incidence, and susceptibility to EMB toxicity. This study includes 131 total human studies from 17 articles, but only eight studies that held in the low-middle income country (LMIC), while the rest is research conducted in developed countries with high incomes. Personalized medicine practices are highly recommended to maintain and obtain the optimal therapeutic effect of EMB.

## 1 Introduction

Tuberculosis (TB) has high mortality rate of infectious disease in the world, affecting an estimated ten million people per year in 2019. According to the World Health Organization (WHO), tuberculosis (TB) is one of the top 10 causes of death globally, and in 2020, an estimated 10 million people fell ill with TB and 1.5 million died from the disease [(WHO) World Health Organization, 2020a]. In contrast, atypical mycobacterial infections are generally less severe and less widespread, although they can still cause significant morbidity and mortality in certain populations, such as those with weakened immune systems (Falkinham, 2015; Drummond and Kasperbauer, 2019; Winburn and Sharman, 2022). The reason of the importance to handle the *Mycobacterium tuberculosis* (Mtb) is based on recent article which highlights the ongoing challenges in TB control and the need for new tools and strategies to combat the disease. This article underscores the continued global burden of TB and the urgent need for new approaches to prevention, diagnosis, and treatment (Esmail et al., 2022).

Most drug-susceptible (DS)-TB patients will have a positive treatment response when treated with the right combination of first-line anti-TB drugs and treatment regimen (duration and dose of the drugs). The World Health Organization (WHO) stated that ensuring access to effective and optimal treatment is a key component to eradicate TB through the End TB Strategy, which includes a priority indicator that 90% or more patients should have a successful treatment outcome (World Health Organization, 2020b). According to WHO, high burden countries for TB are consist of 30 Low- and Middle-Income Countries (LMIC) (World Health Organization, 2021). Ongoing delayed of diagnosis and treatment in LMIC still become problems, worsening prognosis and also continuing TB transmission in the community (Teo et al., 2021). Furthermore, this condition is detrimental to resistance of TB therapy. Resistance to the therapy and risk of drugs adverse effects are the things that could detain to achieve optimal therapy (Pradipta et al., 2018). So, personalized treatment that depends on the individual genetic profile can minimize the risk of toxicity and resistance, thus optimize the treatment. It becomes necessary because of the diversity of drug responses related to the variation in human DNA, such as single-nucleotide polymorphisms (SNPs), which are a single substitution of nucleotides for another (Lander et al., 2001; Subramanian et al., 2001; Kurkó et al., 2013). SNPs that occur in genes related to pharmacokinetics (PK) and pharmacodynamics (PD) processes could affect the response, effectiveness, resistance, and toxicity of drugs (Zastrozhin et al., 2018; Calcagno et al., 2019; Xiang et al., 2020).

Ethambutol (EMB) is a drug widely used for TB treatment. It is used as the first-line anti-TB drug together with rifampicin (RIF), pyrazinamide (PZA), and isoniazid (INH) as a six-month regimen. EMB should not be used alone as monotherapy but rather in tandem with at least one other anti-TB drug. EMB shows a specific effectiveness against the Mtb and atypical/non-tuberculous mycobacteria such as *Mycobacterium avium complex bacteria* (MAC) that cause pulmonary infection non-tuberculosis and lymphadenitis, but not against other bacteria or other pathogens, such as viruses and fungi (Lee and Nguyen, 2020; Winburn and Sharman, 2022) EMB is also a potential supplementary agent for a treatment regimen of multidrug-resistant (MDR) and extensively drug-resistant (XDR) TB (World Health Organization, 2020a). Although as a supplementary agent, EMB is added to the TB regimen as a protection against unrecognized resistance to one of the three core drugs (Horsburgh et al., 2015). However, the WHO recently reported that patients infected with *Mtb* strain and showing simultaneous resistance to EMB and INH or EMB and RIF had an increased risk of treatment failure and further acquired resistance (Falzon et al., 2011). EMB is a bacteriostatic drug that interferes with the biosynthesis of arabinogalactan in the cell wall of *Mtb* and inhibits multiplication by bacilli (Palomino and Martin, 2014). However, the exact molecular mechanism of action remains unclear (Schubert et al., 2017). Studies on genetic polymorphisms related to EMB thus far have focused on polymorphisms that occur in the gene of *Mtb* bacteria (Zenteno-Cuevas et al., 2019). Only a few studies have explored gene polymorphisms in humans that have an impact on the clinical response to EMB, including resistance and toxicity.

In human genetic polymorphism studies of anti-TB, the majority focus on RIF or INH. These studies discussed polymorphisms related to clinical response and toxicity (Baskaran and Sabina, 2017; Bao et al., 2018; Richardson et al., 2018; Yang et al., 2019; Zenteno-Cuevas et al., 2019; Rivière et al., 2020). However, EMB has an important role in the treatment of tuberculosis, such as maintaining the effectiveness of therapeutic regimens (Horsburgh et al., 2015). In addition, the incidence of EMB toxicity is lower than that of other first-line anti-TB drugs, especially in comparison with INH. The toxicity is also thought to be related to polymorphisms in certain drug-metabolizing enzymes (DMEs) (Castro et al., 2015; Sarkar and Ganguly, 2016).

The relatively low toxicity and low incidence of resistance are the reasons why EMB is widely used in both sensitive and resistant TB. Personalized medicine practices are highly recommended to maintain and obtain the optimal therapeutic effect of EMB, but human studies of polymorphisms related to EMB clinical response, which is the basis for personalized medicine-based therapy, are scarce. Mapping out all the polymorphisms that have an impact on EMB efficacy, risk of toxicity, and risk of resistance can help us maintain the effectiveness of EMB as one of the TB drugs for sensitive and resistant TB. This article summarizes the genetic polymorphisms associated with alterations in the pharmacokinetic profile, resistance incidence, and susceptibility to EMB toxicity.

## 2 Materials and methods

**FIGURE 1:**
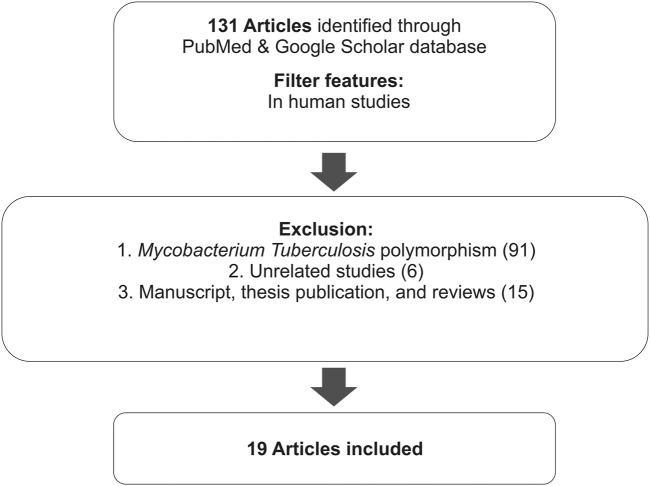
Research flow.

This review summarizes the results of several studies related to the effects of polymorphisms on the EMB clinical response (effectivity, pharmacokinetics, resistance, and susceptibility to toxicity). It includes studies from the PubMed database identified using the keywords “genetic polymorphism” and “ethambutol”. Furthermore, research communications, thesis manuscript, reviews, expert opinions, non-English studies, and unrelated studies were excluded (Figure 1). The thesis manuscript that we have found is the manuscript who have not already been published, and article reviews that were excluded are articles in the form of narrative reviews or mini-reviews that do not contain statistically new conclusions.

A total of 131 reports of human studies were collected; among them, 91 studies of *Mtb* polymorphism, 11 review studies, 4 thesis publications, and 8 unrelated studies, which discuss genetic polymorphism related to susceptibility of TB, were excluded. Hence, a total of 17 articles were included in this study. Most of the articles discussed the effect of gene polymorphisms on the therapeutic outcome, i.e., effectiveness, resistance to EMB, and side effects or toxicity.

## 3 Result

### 3.1 Ethambutol

EMB (C10H24N2O2) is an ethylenediamine derivative used as the dihydrochloride salt. It is a white crystalline powder that is essentially odorless and has a bitter taste. It is sparingly soluble in water (Osol and Hoover, 1976). EMB was first discovered at Lederle Laboratories of the American Cynamid. The discovery of its remarkable stereospecific activity in mice was found in 1961 (Thomas et al., 1961; Shepherd et al., 1966).

#### 3.1.1 Ethambutol and tuberculosis

Early biochemical studies found that EMB works by impairing glycerol metabolism as well as RNA synthesis only on bacilli (Kuck et al., 1962; Kuck et al., 1963; Kuck et al., 1965). Subsequent biochemical studies showed that EMB interferes with the biosynthesis of arabinogalactan, a major component of the bacterial cell wall. The polymerization of cell wall arabinan from arabinogalactan and lipoarabinomannan is inhibited by blockade of arabinosyl-transferases and enchance the accumulation of D-arabinofuranosyl-P-decaprenol, an intermediate in arabinan biosynthesis (National Center for Biotechnology Information, 2021). This results in halting bacterial growth. Genetically, the effect of EMB is related to interactions with three membrane-embedded *arabinosyltransferas*e genes, *EmbA, EmbB, and EmbC*. Most of pharmacogenomics study on EMB discussing about these genes. EMB has a specific mechanism as a therapy for tuberculosis (Palomino and Martin, 2014; Lee and Nguyen, 2020; Zhang et al., 2020; National Center for Biotechnology Information, 2021). For DS-TB, EMB is used together with other first-line anti-TB drugs, RIF, INH, and PZA, and for drug-resistant (DR)-TB, it is used in combination with other second-line anti-TB drugs. For MDR TB and rifampicin-resistant tuberculosis (RR TB), it is recommended to use EMB with delamanid, PZA, imipenem-CILAStatin/meropenem, amikacin/streptomycin, ethionamide/prothionamide (Pto), and p-amino salicylic acid (Lee and Nguyen, 2020).

A previous study showed that EMB has a synergistic effect with INH against *Mtb* through a transcriptional repressor of the inhA gene, a target gene of INH that encodes a protein for cell wall integrity. The results indicated that EMB enhances INH sensitivity of the inhA gene and, as a result, might increase the killing effect and toxicity of INH (Zhu et al., 2019). The WHO updated treatment guidelines for DR TB in May 2016 that recommend use of the shorter MDR-TB regimen under specific conditions, including use of high-dose INH and EMB. The addition of high-dose INH or EMB (or both) strengthens the regimen. This new recommendation is expected to be more effective and provide more benefit for most MDR-TB patients worldwide [(WHO) World Health Organization, 2020b].

#### 3.1.2 Pharmacokinetics and pharmacodynamics

**FIGURE 2:**
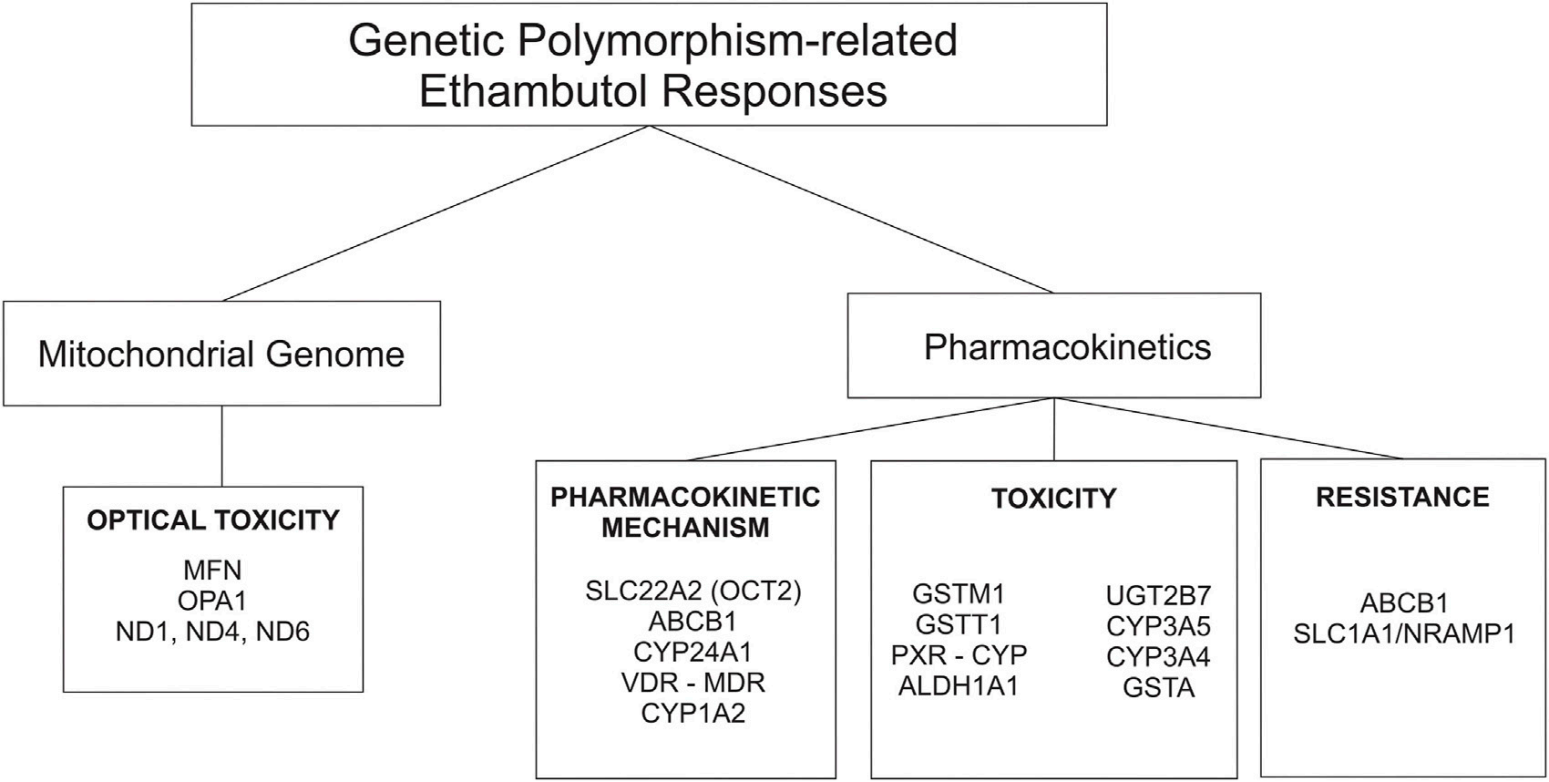
Genetic polymorphisms-related ethambutol responses on pharmacokinetic mechanism and mitochondrial genome.

Approximately 75%–80% of an orally administered dose of EMB (hydrochloride form) is absorbed in the gastrointestinal tract. Absorption is not substantially affected when the drug is administered with food. EMB is widely distributed in most body tissues and fluids. The highest concentrations of the drug are found in erythrocytes, kidneys, lungs, and saliva; lower drug concentrations are found in ascitic fluid, pleural fluid, brain, and Cerebrospinal Fluid (CSF). In tuberculosis meningitis, only 10%–50% of EMB may penetrate the meninges (Mc Evoy, 2007; United States Pharmacopeial Convention, 2007; World Health Organization, 2020a). The absorption and distribution profile of the drug is affected by many enzymes, and those that play a major role are transporter enzymes, such as the ATP-binding cassette (ABC transporter) and solute carrier family (SLC transporter) (Figure 2) (Parvez et al., 2017; Pontual et al., 2017).

Half of EMB is excreted unchanged in the urine, and a small part is excreted in the feces. An additional 15% changed to the form of metabolites. EMB is partially inactivated by oxidation to an aldehyde intermediate, which is converted to the decarboxylic acid derivative assisted by a metabolic enzyme called *aldehyde dehydrogenase 1* (ALDH1) (Figure 2) (PEETS et al., 1965; Peng et al., 2021). Other drug-metabolizing enzymes (DMEs), such as c*ytochrome* (CYP), also play a role in the metabolic rate of EMB. Based on a recent study, the AUC_0-8_ varied from 6.3 ± 5.5 h mg/L to 10.8 ± 7.6 h mg/L depending on *CYP1A2* genetic polymorphisms (Sundell et al., 2020). In addition, the plasma half-life of EMB is approximately 3.3 h in patients with normal renal function. The half-life is prolonged in patients with impaired renal or hepatic function. In patients with renal failure, the half-life can be 7 hours or longer (Mc Evoy, 2007).

EMB is a concentration-dependent bactericidal, where ability to kill bacteria is linked to AUC/MIC. Decreasing the AUC could lead to decreased killing activity and increase the risk of treatment failure (Hall et al., 2012). Therefore, polymorphisms in genes that code for enzymes that play a role in drug pharmacokinetics, will also affect the drugs response. genetic polymorphisms that occur in genes encoding enzymes that play a role in pharmacokinetic processes in turn affect pharmacodynamics processes.

#### 3.1.3 Toxicity and resistance of EMB

One of the most well-known and major adverse effects of EMB is optic neuropathy. It is dose-related, and 40% of adult patients develop optic neuropathy at doses greater than 50 mg/kg (Donald et al., 2006). Non-etheless, EMB is included in the category of TB drugs with mild adverse effects (World Health Organization, 2020b). A previous study conducted on 60 pulmonary TB patients treated with EMB resulted in significant changes in copper (Cu) levels. The study find that the chelating effect of EMB could leads to the decrease in serum levels of Cu as cationic trace elements. This increases the chelation of copper, which is related to the mechanism of EMB-induced optic neuropathy. Blood samples were obtained before treatment (baseline) and 10 days after starting anti-TB therapy, and the amounts of serum Cu were determined in all samples by atomic absorption (Abbasi Nazari et al., 2009). Another factor that could be a susceptible factor for optic neuropathy is genetic polymorphism of the human-mitochondrial gene. Mitochondrial genes encode proteins that play a role in cell survival and can decay as a result of alterations in genetic sequences, such as the presence of SNPs (De Marinis, 2001; Seo et al., 2010) (Figure 2). Apart from the major toxicity of EMB (reversible-optical nerve degradation), EMB may play a role in hepatotoxicity. Some studies found that EMB has adverse effects on hepatotoxicity, but others do not (Ramappa and Aithal, 2013). The fact that genetic polymorphism could increase the susceptibility of EMB toxicity, including hepatotoxicity, is known based on previous studies (De Marinis, 2001; Seo et al., 2010; Ramappa and Aithal, 2013; Fatiguso et al., 2016).

Genetic polymorphisms could be a potential marker of EMB resistance (Ramachandran and Swaminathan, 2012; McCallum and Sloan, 2017; Motta et al., 2018). EMB is added to the TB regimen as protection against unrecognized resistance to one of the three core drugs (Horsburgh et al., 2015). But, as mentioned before, the majority of studies found that the mechanism of resistance to EMB was linked to mutations in the gene *embB* with mutations at position *embB306* (Sreevatsan et al., 1997; Telenti et al., 1997). The primary resistance rates of *Mtb* to EMB vary widely from 1% to 14% (Nasiri et al., 2016). Patients infected with *Mtb* strains showed simultaneous resistance to EMB and INH or EMB and RIF, which has been associated with an increased risk of treatment failure and further acquired resistance (Falzon et al., 2011).

Other possible mechanisms of drug resistance related to the host, human genetic polymorphisms, are SNP on the genes that play a role for pharmakokinetics and pharmacodynamic of EMB (Figure 2).

### 3.2 Pharmacogenomics

The diversity of drug responses was revealed with the completion of the Human Genome Projects in April 2003. A developing field called personalized medicine has adapted medical care, such as treatment decision-making, to the genetic background of individuals. Pharmacogenomics typically involves the search for variations in multiple genes that are associated with variability in drug response. Understanding genetics of an individual is the key to creating personalized drugs that will optimize therapy (National Human Genome Research Institute, 2018).

#### 3.2.1 Genetic polymorphism related to pharmacokinetic mechanism

**FIGURE 3:**
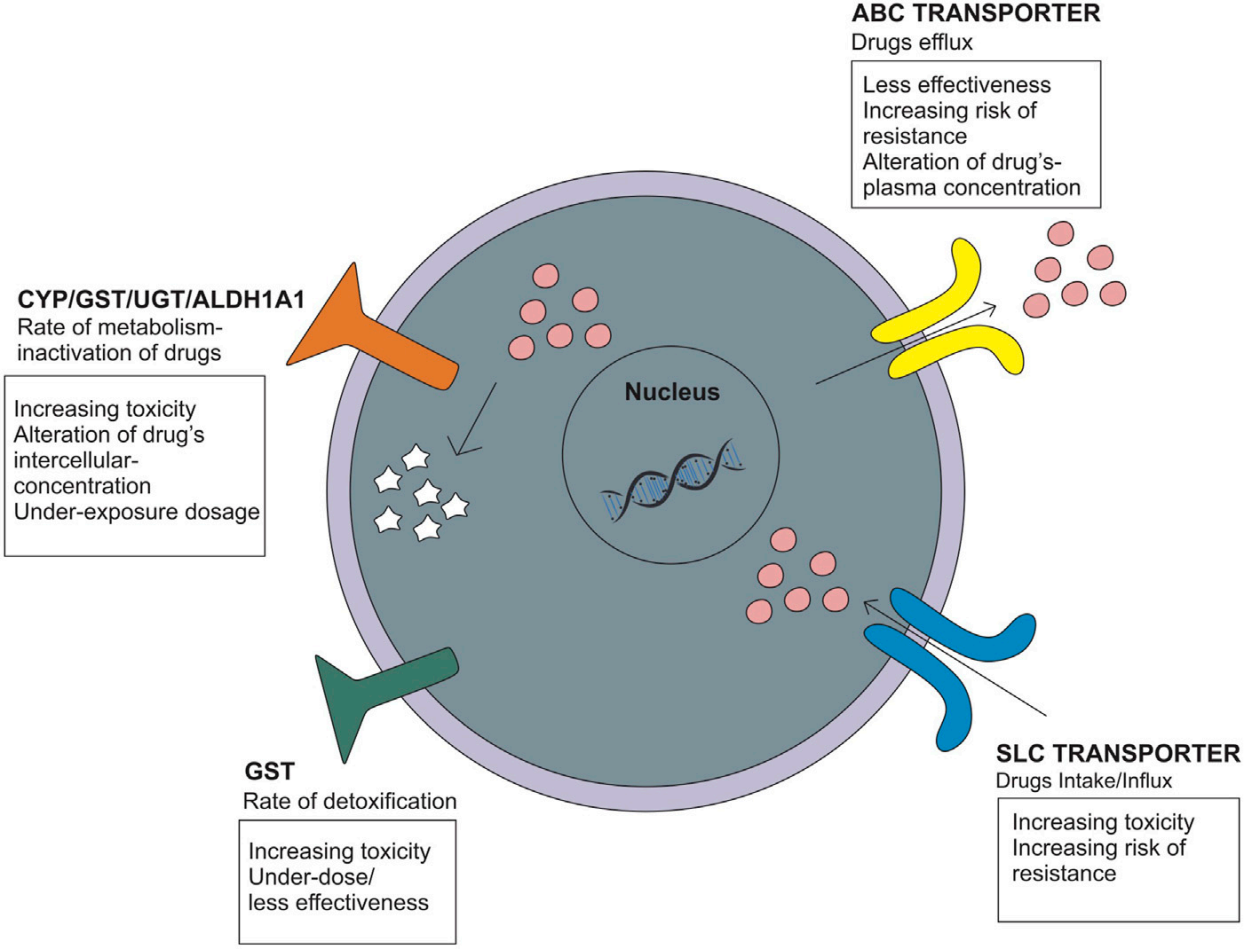
Genetic polymorphisms related to the pharmacokinetic mechanism that influences the drug intake/efflux and the rate of drug metabolism.

Genetic polymorphisms can change the pharmacokinetic profile of EMB, such as creatinine clearance, plasma concentration, and drug exposure, and result in the possibility of toxicity or drug resistance (Table 1). Genes that are included in this category are genes that encode *ATP-binding cassette* (ABC transporter), *solute carrier family* (SLC transporter), metabolizer enzymes; *cytochrome* (CYP), *UDP-glucuronosyltransferase* (UGT), *aldehyde dehydrogenase* (ALDH), *glutathione S-transferase* (GST), *vitamin D receptor* (VDR), *pregnane X receptor* (PXR), and genes related to optic neuropathy (Takahashi et al., 2008; Ramachandran and Swaminathan, 2012; Fatiguso et al., 2016; Pontual et al., 2017; Sun et al., 2019; Peng et al., 2021) (Figure 3).

**TABLE 1:** Genetic polymorphism related to pharmacokinetic mechanism.

Genes	Polymorphism	Subject	Country	LMIC	Conclusion	Ref
ABCB1	rs1128503	218 patients	Brazil	Middle	This study demonstrated that inter-individual variability in anti-TB treatment with P-gp substrate drugs is clinically relevant	Pontual et al. (2017)
	rs2032582					
	rs1045642					
ABCB1	rs1045642	24 patients	Italy	High	Possible role of single nucleotide polymorphisms on EMB plasma and intracellular concentrations	Fatiguso et al. (2016)
VDR	rs731236					
	rs7975232					
	rs10735810					
	rs1544410					
	rs11568820					
CYP24A1	rs927650					
	rs2585428					
	rs2248359					
CYP27B1	rs4646536					
	rs10877012					
OCT2	rs201919874	Human Embryonic Kidney Cells (*in vitro*- *in vivo* extrapolation)	South Korea	High	This *in vitro* to *in vivo* extrapolation study showed genetic polymorphism in OCT2 transporter affected on EMB pharmacokinetics which may explain inter-individual response	Parvez et al. (2017)
	rs316019					
	rs145450955					
SLC11A1	rs34448891	95 patients	Japan	High	Genetic variations in SLC11A1 may affect the incidence of MDR-TB and clinical features of pulmonary tuberculosis	Takahashi et al. (2008)
	rs3731865					
	rs17235409					
	rs17235416					
GSTM1		451 studies	China, Brazil, India, Spain, Korea, Japan	Middle - High	This meta-analysis provides evidence that there may be an increased risk of ADIH in individuals with null genotypes of GSTM1 in the total population, especially East Asians and patients receiving HRZE or HRZES.	Li et al. (2013)
GSTT1						
ALDH1A1	rs3764435	747 patients	China	Middle	rs7852860 variants in ALDH1A1 gene is associated with susceptibility to ATDILI in the Chinese Han population	Peng et al. (2021)
	rs348471					
	rs63319					
	rs610529					
	rs7027604					
	rs8187876					
	7852860					
CYP3A4	rs2242480	297 patients	China	Middle	In summary, CYP3A4*18B-20232G/A, UGT2B7-268A/G, and UGT2B7 802C/T wild-type genotypes and CYP3A5*3-6986A/G mutant genotypes are related to the development of ADIH for TB patients receiving anti-TB chemotherapy	Sun et al. (2019)
UGT2B7	rs7662029					
	rs7439366					
CYP3A5	rs776746					
CYP1A2	rs142777869					
CYP2C19	rs4244285					
GSTA1	rs3957356					
GSTM3	rs1799735					
NAT2	rs1799930	221 patients	Uganda	Low	On HIV/Tuberculosis Coinfected patients, genetic polymorphisms on PXR, SLCO1B1, and NAT2 were moderately associated with INH exposure (pharmakokinetic), whereas PXR rs2472677 with T allele showed worse outcomes such as higher risk of death	Calcagno et al. (2019)
SLCO1B1	rs4149032					
PXR	rs2472677					
CYP2E1	rs6413432	28 patients	China, Taiwan, Korea, Japan, India, Tunisia, Brazil, Canada	Middle-High	The study observed significant associations between the RsaI and 96-bp deletion-insertion SNPs of the CYP2E1 gene and anti-tuberculosis drug-related hepatotoxicity	Richardson et al. (2018)
CYP2E1	rs6413432	54 studies	China, Korea, India, Tunisia, Turkey, Japan, Taiwan, Iran, Brazil, Indonesia, Thailand	Middle - High	ATDILI is more likely to occur in patients with NAT2 slow acetylator genotype, CYP2E1 RsaI/PstI c1/c1 genotype and GSTM1 null genotype	Yang et al. (2019)
NAT2	rs1799930					
GSTM1						
GSTT1						
SLCO1B1	rs2306283					
	rs4149056					
CYP1A2	rs2069514	63 patients	Rwanda	Low	There are a significant result about the association between SNP and 50% reduction in relative bioavailability. CYP1A2 polymorphism might affects the ethambutol exposure	Sundell et al. (2020)

##### 3.2.1.1 ABC transporter and SLC transporter

The *ATP-binding cassette* (ABC transporter) and *solute carrier family* (SLC transporter) genes encode protein transporters that play a role in the efflux and influx/uptake of drugs into cells (Figure 2). Polymorphisms of both genes may alter the activity or function of proteins and have an impact on drug concentration at the target site, leading to alterations in therapeutic efficacy or risk of resistance. Polymorphisms in *ABC transporters*, especially *ABCB1* encoding P-glycoprotein (P-gp), a transmembrane drug efflux pump, may contribute to drug resistance and/or drug effectivity. The *ABCB1* rs2032582 AA genotype is specifically associated with EMB resistance. A case–control study in Brazil showed that analyses for EMB resistance revealed an association of the rare allele “A” (OR = 12.91 *p* = 0.01) (Pontual et al., 2017). The wild-type genotype of *ABCB1* is CC, which encodes P-gp and has a physiological function to remove toxic metabolites. Polymorphism in *ABCB1* rs1045642 is associated with alteration of EMB plasma concentration with the result of homozygote mutant and heterozygote genotype (TT and CT) on week two and week four has a significant association with plasma concentration through *p* = 0.023 and *p* = 0.035, respectively (Fatiguso et al., 2016). Meanwhile, the SLC transporter is a transmembrane influx/uptake pump. Like *ABC transporters*, polymorphisms of *SLC transporters* may contribute to drug resistance and/or drug effectivity. An *in vitro* to *in vivo* extrapolation study showed that a genetic polymorphism in the *SLC22A2* (OCT2) gene affected the EMB pharmacokinetics profile. Mutant genotypes reduced the activity of EMB transport and affect the drug clearance rate and AUC value (Parvez et al., 2017). Another study of *SLC11A1/NRAMP1* (natural resistance-associated macrophage protein 1) in patients who used INH, PZA, RIF, EMB, kanamycin, and streptomycin showed that genetic variations in *SLC11A1* may affect the incidence of MDR-TB (OR = 5.03 95% CI [1.24-20.62] *p* = 0.02 and OR = 5.03 CI95% [1.24-20.62] *p* = 0.02) and clinical features of pulmonary TB, which could be explained by a longer time to sputum culture conversion. EMB most likely contributed to these statistical results because G alleles are likely related to EMB resistance (Takahashi et al., 2008).

##### 3.2.1.2 Cytochrome (CYP), UDP-glucuronosyltransferase (UGT), aldehyde dehydrogenase (ALDH), and glutathione S-transferase (GST)

Genes that encode metabolizer enzymes, such as *CYP*, *GST*, *UGT*, and *ALDH*, contribute to drug resistance and have been widely studied. Drugs metabolized for the two purposes; converting drugs into an active form and/or inactive drugs so that they can be excreted through the kidney. Changes in the function/activity of drug-metabolizing proteins will also affect the level of the active form of the drug at the target site (Susa and Preuss, 2021). Several studies have been performed to analyze the association between *CYP* polymorphisms and EMB responses. *CYP24A1* rs2585428 is associated with EMB plasma and intracellular concentrations. The AG and GG genotypes of *CYP24A1* rs2585428 had a significant association with the decrease of intracellular Ctrough of EMB (*p* = 0.03) (Fatiguso et al., 2016). Another study conducted in HIV-TB patients with the *CYP1A2* rs2472304 polymorphism showed that the GA genotype was associated with a 50% reduction in EMB bioavailability. This SNP affects EMB exposure, and the treatment given may result in underexposure dosage (Sundell et al., 2020). Furthermore, *GST* polymorphisms are related to susceptibility to drug toxicity. GST are recognized as common detoxifying enzymes, playing an important protective role as they catalyze the conjugation of various reactive drug toxicity metabolites causing cellular damage with glutathione, thereby decreasing drug hepatotoxicity (Mitchell et al., 1973; Meister, 1983; Meister and Anderson, 1983; Andreoli et al., 1986; Li et al., 2013). The *GSTM1* rs4025935 null genotype is associated with increased anti-tuberculosis drug-induced hepatotoxicity (ATDIH) (OR = 1.36 CI 95% [1.04-1.79]). Pooled analysis result null allele carriers had significant association with ATDIH risk for INH, RIF, PZA, and EMB regimen (OR = 1.47 CI 95% [1.14-1.9] *p* = 0.406) and INH, RIF, PZA, EMB, and streptomycin regimen (OR = 1.89 CI 95% [1.09-3.27] *p* = 0.076). However, there was no significant association in the dual therapy INH and RIF-only regimens (Li et al., 2013).

*Aldehyde dehydrogenase 1 family member A1* (ALDH1A1) is the next enzyme after alcohol dehydrogenase in the major pathway of alcohol metabolism (Wu et al., 2020; NCBI, 2021). In addition to alcohol, many drugs can be metabolized by ALDHs, such as EMB, and its L-isomer is metabolized by liver ALDHs to form an aldehyde (oxidative) metabolite. The oxidation of aldehydes is generally considered a detoxification process, and a decrease in the rate of the mechanism could affect the risk of toxicity (Wu et al., 2020). The association between the *ALDH1A1* polymorphism and incidence of anti-TB drug-induced liver injury (ATDILI) was investigated (Chang et al., 2018; Zhong et al., 2021). The results showed that rs7852860 variants in the *ALDH1A1* gene are associated with susceptibility to ATDILI. The C allele and the CA genotype of rs7852860 were significantly associated with an elevated risk for ATDILI (*p* = 0.006 and 0.005, respectively). Unfortunately, there were limitations of the study in that it was only conducted on Chinese people with a small number of samples (Peng et al., 2021). Another gene related to the hepatotoxicity of EMB and the anti-TB regimen is *UGT*. Members of the *UGT* family probably make the largest contribution to phase II metabolism of drugs implicated in DILI, and it is relevant because of their role in detoxifying reactive metabolites. Loss of function of the *UGT* gene could increase the active drug level that cannot be excreted out of the body, thereby increasing the risk of toxicity (Daly, 2017). A study on *UGT2B7* rs7662029 found that the AG genotype could be a protective factor against ATDILI (*p* = 0.00 OR = 0.389) (Sun et al., 2019).

##### 3.2.1.3 Vitamin D receptor (VDR) and pregnane X receptor (PXR)

*VDR*, a ligand-activated transcription factor, controls gene expression. The active form of vitamin D, 25-hydroxyvitamin D, binds to *VDR*, controlling the synthesis of many different proteins (Abouzid et al., 2018). *VDR* binds directly to specific sequences located near promoters and recruits a variety of coregulatory complexes that perform the additional functions required to modify transcriptional output (Pike and Meyer, 2010). Transcriptional output alteration can influence the production of RNA, which encodes proteins that are integral to specific biological activities such as the degradation of xenobiotic compounds in several tissues (Bouillon et al., 2008; Carlberg and Campbell, 2013) and the functions of key cell types involved in both innate and adaptive immunity (Cutolo et al., 2011). *Pregnane X receptor* (PXR) is another ligand-activated nuclear receptor (NR) that mainly controls inducible expression of xenobiotic handling genes, including biotransformation enzymes and drug transporters (Pavek, 2016). Both *VDR* and *PXR* play major roles as transcription factors of several genes.

*CYP3A4* is considered to be the most important member of the family of drug-metabolizing CYP450 enzymes, contributing importantly to the clearance of therapeutic agents (Guengerich, 1999). The induction of *CYP3A4* gene expression is mainly regulated through activation of *PXR* (Lin, 2006; Wang et al., 2013) and it has been demonstrated that the active form of vitamin D3 (1α,25(OH)2D3) can also enhance the transcription of *CYP3A4* by a VDR-mediated pathway (Wang et al., 2013).

A study on *VDR* BsmI (rs1544410), ApaI (rs7975232), and Cdx2 (rs11568820) suggested a possible role of that SNP in EMB plasma concentration and intracellular concentration (Fatiguso et al., 2016). However, a study on *PXR* rs2472677 showed that the *PXR* polymorphism has a significant correlation with INH exposure but not with EMB and other anti-TB agents (Calcagno et al., 2019).

#### 3.2.2 Genetic polymorphism related to optic neuropathy

Mitochondrial function is influenced by several genes. Polymorphism or mutations in mitochondrial genes affect the ability of cells to survive, which in turn has an impact on nerve degeneration, one of which is the optic nerve (Table 2).

**TABLE 2:** Genetic polymorphism related to optic neuropathy.

Genes	Subject	Country	LMIC	Conclusion	Ref
OPA1	10 patients (skin fibroblast, *in vitro*)	Italy	High	The results disclose a novel link between OPA1, apoptosis inducing factor and the respiratory complexes that may shed some light on the pathogenic mechanism of DOA (Dominan Optic Atrophy)	Zanna et al. (2008)
OPA1	1 patient	France	High	In fibroblasts from the patient carrying OPA1_p.I382M mutations, EMB treatment did not lead to any additional reduction of complex IV activity. However, Mitochondrial genetic variations may therefore be predis- posing factors in EMB-induced ocular injury	Guillet et al. (2010)
mt-ND	3 cases from 46 total subject	Korean	High	Anti-tuberculosis medication may be an epigenetic factor of LHON in patients with a primary LHON mutation	Seo et al. (2010)
mt-ND	1 patients	Italy	High	They believe that their patient had EMB optic neuropathy. It cannot be excluded that the heteroplasmic DNA mutation of LHON may have predisposed the patient to toxic neuropathy	De Marinis (2001)
mt-ND	2 patients	United Kingdom	High	Decreased levels of mtDNA-encoded ND1 and several nuclear encoded complex I subunits in both cases	Ng et al. (2020)
MFN2/CMT2A2	1 patients	United States	High	This case shows that patients with CMT2A2, and possibly other mitochondrial fusion defects, may be uniquely susceptible to ethambutol-induced neurotoxicity	Fonkem et al. (2013)

##### 3.2.2.1 Mitofusion (MFN) and optic atrophy 1 (OPA1) protein

**FIGURE 4:**
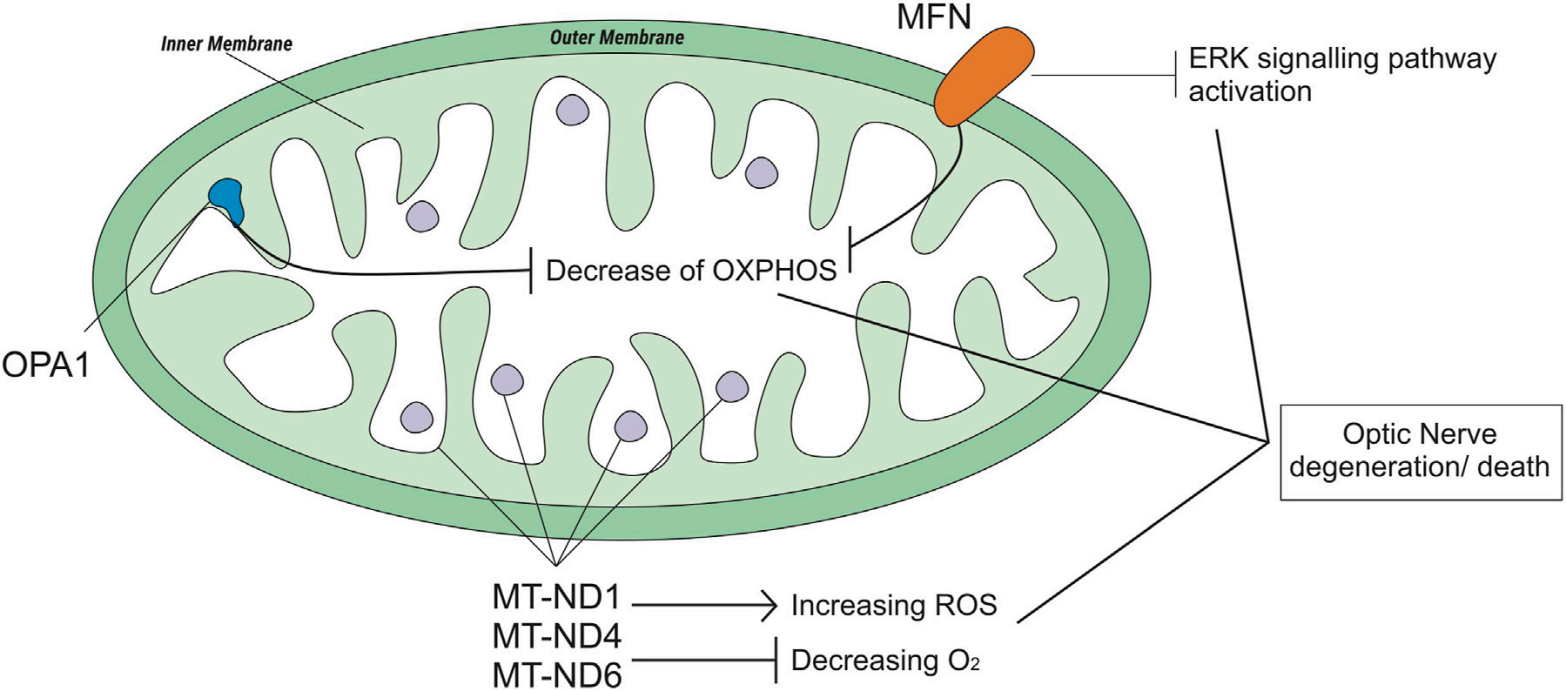
Genetic polymorphisms related to optic neuropathy which is regulated by the mitochondrial genome.

Mitochondria are cell organelles (mitochondrion, singular) that generate most of the chemical energy needed to power the biochemical reactions of cells (William Gahl, 2021). It plays an important role in cell function and survival (Sedlackova and Korolchuk, 2019). Mitochondrial fusion and fission are mechanisms that promote mitochondrial health and survival via the exchange of mitochondrial proteins, lipids, and genomes. Mitochondrial fusion is mediated by proteins called MFN1 and MFN2 on the outer mitochondrial membrane and OPA1 on the inner mitochondrial membrane (Liesa et al., 2009; Chen et al., 2011) (Figure 4). The *MFN* gene encodes the mitofusin protein, a protein that helps determine the shape and structure of mitochondria, the energy-producing centers organelle within cells (Chen, Liu and Dorn 2nd, 2011). *OPA1* is involved in a process that takes place in mitochondria called oxidative phosphorylation, from which cells derive much of their energy, play a role in the maintenance of the DNA within mitochondria (mtDNA), and are also involved in apoptosis mechanisms (Zanna et al., 2008; Yu-Wai-Man et al., 2010). Loss of function of these mitochondrial fusion proteins (caused by mutations) could lead to degenerative neurological disease (Liesa et al., 2009), such as autosomal dominant optic atrophy (DOA), which is induced by *OPA1* mutation (Yu-Wai-Man et al., 2010), and *Charcot-Marie-Tooth* (CMT), which has a subacute onset of optic atrophy associated with central scotoma and color vision defects (Guerriero et al., 2020).

The most common form of axonal *CMT* (CMT2) is *CMT2A2*. A study on *CMT2A2* cases showed that patients with *MFN2* polymorphisms and possibly other mitochondrial fusion defects may be uniquely susceptible to EMB-induced neurotoxicity (Fonkem et al., 2013). Otherwise, a case study on the *OPA1* rs143319805 polymorphism showed that the basal respiratory rate of the mutant was higher than that of the controls/wild type (Guillet et al., 2010).

##### 3.2.2.2 NADH-ubiquinone oxyreductase core (ND)

The *mitochondrial NADH-ubiquinone oxidoreductase core* (*MT-ND*) encodes a NADH dehydrogenase protein, a part of a large enzyme complex known as complex I. *MT-ND* is activated in mitochondria and plays an important role in producing energy through a process called oxidative phosphorylation, which uses oxygen and simple sugars to create adenosine triphosphate (ATP), the main energy source of the cell. There are several types of *MT-ND* genes, *MT-ND1*, *MT-ND4*, *MT-ND6*, and polymorphisms in these genes lead to biochemical alterations (Lenaz et al., 2004). Mitochondrial complex I deficiency is also associated with a diverse range of clinical phenotypes (Ng et al., 2020), such as Leber hereditary optic neuropathy (LHON). LHON is a maternally inherited form of central vision loss/degeneration associated with mitochondrial DNA point mutations that affect the ND subunits of the complex (De Marinis, 2001; Lenaz et al., 2004; Seo et al., 2010). A case report from Korea stated that anti-TB medication, specifically EMB, could be a possible epigenetic factor of LHON (Seo et al., 2010). Another case report of subjects with LHON suggested that EMB could have acted as a trigger factor for LHON (De Marinis, 2001).

## 4 Discussion

From this study we got only eight studies (42.1%) that held in the LMIC, while the rest is research conducted in developed countries with high incomes. In contrast, globally more than 90% of reported tuberculosis infections occur in LMIC. Despite a cumulative reduction in global tuberculosis-related deaths, global progress is still far away from the targets set by the WHO on End TB Strategy and the United Nations on Sustainable Development Strategy. Goals on tuberculosis (SDG 3.3) (WHO, 2015; 2020; UN, 2022), efforts are needed to increase the optimization of tuberculosis therapy, especially in LMIC.

EMB is widely used in both sensitive TB and resistant TB (Lee and Nguyen, 2020) cases. A global systematic and meta-analysis stated that the resistance and the increasing risk of toxicity were a trend towards increase risk of MDR-TB (Pradipta et al., 2018). It is well known that resistance and risk to toxicity might be affected by the genetic polymorphism. Although only a few studies have explored the association between human genetic polymorphisms and EMB clinical response, we hypothesize that genetic polymorphisms influence the clinical response to EMB by two mechanisms: 1) alteration of the pharmacokinetic profile leads to ineffective therapy due to resistance and an increased risk of drug toxicity, and 2) alteration of the expression or activity of mitochondrial-related genes may lead to an increased risk of EMB optic toxicity. EMB is included in the category of TB drugs with mild adverse effects (World Health Organization, 2020a). The toxicity of EMB is mainly reversible optical nerve degradation, but as a regiment, EMB might also play a role in hepatotoxicity. Some studies have shown that EMB has a hepatotoxic effect, but others have not. It is dose-related; more than 40% of adults develop ototoxicity at doses greater than 50 mg/kg (Donald et al., 2006). EMB binds to TetR, a transcriptional regulator that enhances the INH sensitivity of the *inhA* gene and leads to increases in the killing effect of INH, thus increasing INH toxicity (Zhu et al., 2019). Genetic polymorphisms in drug metabolic enzyme-encoded genes, such as *GST*, *CYP*, and *UGT*, could affect the risk of EMB hepatotoxicity (Li et al., 2013; Richardson et al., 2018; Sun et al., 2019). Meta-analysis study which conducted on various middle-and high income countries prove that there are significant association between RsaI and 96-bp deletion-insertion SNPs of the *CYP2E1* gene to the hepatotoxicity (Richardson et al., 2018). The results of a study on the *GSTM1* gene polymorphism (null genotype) showed that the polymorphism was associated with hepatotoxic risk in the EMB-containing regimen, while the non-EMB-containing regimen showed insignificant results. In the *GSTT1* gene polymorphism, the null genotype was associated with hepatotoxic risk in the regimen without EMB. However, previous studies have shown that EMB as monotherapy causes rare or no liver toxicity (Richardson et al., 2018). Another study from China resulted in the possibility that EMB induces/increases the risk of hepatotoxicity when used together with other anti-TB drugs. Unfortunately, the study was only conducted on Chinese people with a small number of samples (Peng et al., 2021). However, this information may add to the evidence that the toxicity of EMB to the liver needs to be considered.

For optical toxicity, the mitochondrial genome plays a role in the majority. Mitochondria, important cell organelles, generate most of the chemical energy needed to power the biochemical reactions of the cell (William Gahl, 2021). The health and survival of mitochondria are maintained through fission and fusion mechanisms, which are mediated by *MFN1* and *MFN2* on the outer mitochondrial membrane and *OPA1* on the inner mitochondrial membrane (Liesa et al., 2009; Chen, Liu and Dorn 2nd, 2011). Genetic disorders due to mitochondrial dysfunction are not uncommon, and the majority of these patients will have eye-related manifestations, including visual loss from the optic nerve and retinal disease, that could be irreversible. Defects in mitochondrial genes such as *MFN* and *OPA1* could cause mitochondrial dysfunction that leads to impaired mitochondrial energy production and oxidative stress (Yu-Wai-Man and Newman, 2017). In addition, *mt-ND*, which is a gene on mitochondrial DNA that functions to code for the NADH dehydrogenase protein, is predicted as an epigenetic factor of LHON in patients with a primary LHON mutation. Some case-report studies on Italy and Korea stated that they believe that their patient had ethambutol optic neuropathy. It cannot be excluded that the heteroplasmic DNA mutation of LHON may have predisposed the patient to toxic neuropathy (De Marinis, 2001; Seo et al., 2010). Several studies and case reports conducted in Italy, England, and United States of America have shown that the risk and susceptibility of ototoxicity-related EMB may be related to genetic polymorphisms of these genes (Guillet et al., 2010; Fonkem et al., 2013). Unfortunately, the authors have not found studies related to *mt-DNA*, *MFN*, and *OPA1* conducted in LMIC. Hence, need further pharmacogenetic study on these genes in patients with EMB therapy in LMIC.

Genetic polymorphisms related to pharmacokinetic mechanisms could affect drug exposure and efficacy and lead to drug resistance. In brief, the pharmacokinetic mechanism is divided into four stages: absorption, distribution, metabolism, and excretion. Transporter proteins, such as ABC transporters and SLC transporters, work on the efflux and influx/uptake of drugs into cells. Polymorphism of the genes that encode these proteins affect the absorption, distribution, and excretion of the drugs (Parvez et al., 2017; Pontual et al., 2017). Genetic polymorphisms in *DMEs*, such as *CYP*, *GST*, and *UGT*, affect drug metabolism. All of these genes play a role in altering the pharmacokinetic rate and affect drug concentration exposure at the target site. Hence, it leads to under-treatment if the concentration is below the therapeutic dose or increases the risk of toxicity/adverse effects if the concentration is higher and enters the toxic dose (Fatiguso et al., 2016; Sundell et al., 2020). Long-term effects and under-treatment or non-optimal therapeutic doses increase the risk of drug resistance (Pontual et al., 2017). In addition, the rate of drug clearance, excretion, or detoxification could affect the increased risk of toxicity (Parvez et al., 2017; Richardson et al., 2018; Sun et al., 2019). Individual variations in the clinical response to therapy are known to be influenced by gene polymorphisms, so studies in this regard should be supported and developed. In addition, the practice of personalized medicine, which aims to minimize the rate of resistance, minimize the incidence of toxicity, and increase the effectiveness of treatment, should be recommended for all diseases, especially TB, given that the key to successful treatment of TB is optimal and effective treatment (World Health Organization, 2020b).

The articles included in this review were not limited in period of time due to the limited number of related studies. This review is subject to slight potential bias, including the influence of the personal viewpoints of the author, gaps in literature searching, and selection methods, which may lead to the omission of relevant research.

## 5 Conclusion

Genetic polymorphisms that occur related to the pharmacokinetics process could alter gene expression or its activities that alter drug concentration (decreased or increased). Therefore, this might be related to the treatment outcome (efficacy and safety/toxicity). Susceptibility to the optic toxicity of EMB could be affected by mitochondrial genetic polymorphism. Personalized medicine is an effort to provide individual therapy based on genetic profiles. Personalized medicine can provide a better and more effective treatment for TB that is efficacious, safe, and prevents drug resistance.
